# Alteration of Serum Gal-3 Levels in Endometrium-Related Reproductive Disorders

**DOI:** 10.3390/ijms26041630

**Published:** 2025-02-14

**Authors:** Reka Brubel, Beata Polgar, Laszlo Szereday, Dora Bianka Balogh, Tunde Toth, Szabolcs Mate, Noemi Csibi, Noemi Dobo, Gernot Hudelist, Nandor Acs, Attila Bokor

**Affiliations:** 1 Department of Obstetrics and Gynecology, Semmelweis University, 1088 Budapest, Hungary; dorabiankabalogh@gmail.com (D.B.B.); dr.mate.szabolcs@gmail.com (S.M.); cs.noemi88@gmail.com (N.C.); dobnoemi22@gmail.com (N.D.); acs.nandor@med.semmelweis-univ.hu (N.A.); attila.z.bokor@gmail.com (A.B.); 2 Department of Medical Microbiology and Immunology, Medical School, University of Pecs, 7624 Pecs, Hungary; polgar.beata@pte.hu (B.P.); szereday.laszlo@pte.hu (L.S.); 3 Department of Anatomy, Medical School, University of Pecs, 7624 Pecs, Hungary; toth.tundi206@gmail.com; 4 Department of Gynecology-Center of Endometriosis St. John of God, Hospital St. John of God, 1020 Vienna, Austria; gernot_hudelist@yahoo.de; 5 Rudolfinerhaus Private Clinic and Campus, 1190 Vienna, Austria

**Keywords:** galectin, endometriosis, non-invasive diagnosis

## Abstract

Endometriosis, a benign, chronic gynecological disorder characterized by the presence of endometrial-like tissue outside the uterine cavity, affects 15% of women of reproductive age. Galectins, a family of beta-galactoside-binding proteins, regulate inflammation and autoimmunity and are widely expressed in reproductive tissues. This study aimed to assess Galectin-3 (Gal-3) levels in the serum of patients with endometriosis compared to asymptomatic controls and investigate serum Gal-3 level changes over a one-year follow-up period of patients with endometriosis. To determine the levels of soluble Gal-3 in the serum of women with endometriosis or gynecological tumors as well as healthy controls, a human Gal-3-specific ELISA was used. Our findings revealed significantly elevated serum Gal-3 levels in patients with endometriosis compared with healthy controls. Furthermore, Gal-3 concentrations were markedly higher in patients with malignant gynecological transformation of the endometrium than in patients with or without endometriosis. During the one-year follow-up, patients with endometriosis exhibited a progressive increase in serum Gal-3 levels. These findings highlight the potential of Gal-3 as a biomarker for endometriosis and related gynecological conditions. However, further prospective studies with larger, more representative patient cohorts are needed to validate its clinical value.

## 1. Introduction

Endometriosis is a chronic condition characterized by the presence of endometrial-like tissue outside the uterine cavity. It is a common gynecological disorder, affecting an estimated 190 million women worldwide. Endometriosis is commonly associated with various symptoms, including dysmenorrhoea, dyspareunia, chronic pelvic pain, and infertility [1]. On the other hand, it may present without symptoms and is diagnosed accidentally in 20–25% of the affected women [1,2]. This condition impacts approximately 15% of women in their reproductive years [3]. The limited understanding of the etiology and pathogenesis of endometriosis likely contributes to the ongoing challenge of developing reliable, noninvasive diagnostic tests for the disease. Endometriosis is marked by the abnormal expression of genes and proteins compared with that in the healthy endometrium, including growth factors, integrins, cadherins, and lectins. These molecules are critical in regulating cell migration, invasion, angiogenesis, immune responses, and apoptosis. Such dysregulation may disrupt molecular and cellular processes, promoting the survival, proliferation, and invasion of ectopic endometrial cells and, ultimately, driving disease progression.

Galectins are suspected to play a crucial role in endometriosis [4,5], as supported by previous research data showing the upregulation of various glycan-binding lectins in this inflammatory disease [6]. Galectins are beta-galactoside binding proteins involved in the regulation of inflammation and autoimmunity. They can regulate activated macrophages and B cells and modulate cell adhesion, migration, and chemotaxis. Galectins are present in various reproductive tissues and at the fetal–maternal interface. Among them, Galectin-3 (Gal-3) is one of the lectins expressed by the human endometrium and decidua [4]. To date, research on galectin expression in endometriosis has primarily focused on Gal-1 and Gal-3, analyzed using immunohistochemistry. A study showed that Gal-3 expression is significantly higher in endometriosis lesions than in the eutopic endometrium of women with endometriosis and markedly greater than in the eutopic endometrium of women without the condition [5].

We previously showed that ectopic implants and peritoneal cells express Gal-9 mRNA. Moreover, we showed that patients with endometriosis exhibit significantly higher serum Gal-9 levels than healthy controls. Enzyme-linked immunosorbent assay (ELISA) analysis further revealed that serum Gal-9 levels were markedly elevated in endometriosis and gynecologic control cases compared with healthy individuals. Receiver operating characteristic (ROC) curve analysis demonstrated that serum Gal-9 levels possess a very high diagnostic value, with an area under the ROC curve of 0.973, a sensitivity of 94%, and a specificity of 93.75%. Furthermore, we observed that soluble Gal-9 in the serum is relatively unstable, showing signs of degradation after six months of storage [7].

This prospective case–control study aimed to assess whether serum Gal-3 levels are elevated in patients with endometriosis compared with healthy controls and examine alterations in serum Gal-3 concentrations during the follow-up of patients with endometriosis.

## 2. Results

A total of 77 women were involved in this prospective case–control study. All of these participants underwent diagnostic or operative laparoscopy due to suspected endometriosis. Among them, 11 patients were diagnosed with stage I–II (mild) endometriosis, and 66 suffered from advanced/severe (stages III–IV) disease; 29 patients with severe endometriosis returned for examination after one month, and 18 were present for follow-up after one year.

The demographic data of our cohort are summarized in Supplementary Table S1.

Considering that our controls were women without endometriosis or with asymptomatic endometriosis (*n* = 14) who were anonymous blood donors, their epidemiological and clinical characteristics were not available (Asymptomatic, A-control). The age distribution showed no statistically significant differences between the study groups (*p* > 0.05). Infertility was present at the same rate among patients with severe and mild endometriosis. Among patients with stage III–IV endometriosis, a higher proportion had previous surgery than those with stage I–II. However, the presence of chronic pelvic pain was similar in these groups. The prevalence of autoimmune diseases and insulin resistance did not differ significantly between the study groups.

**Figure 1:**
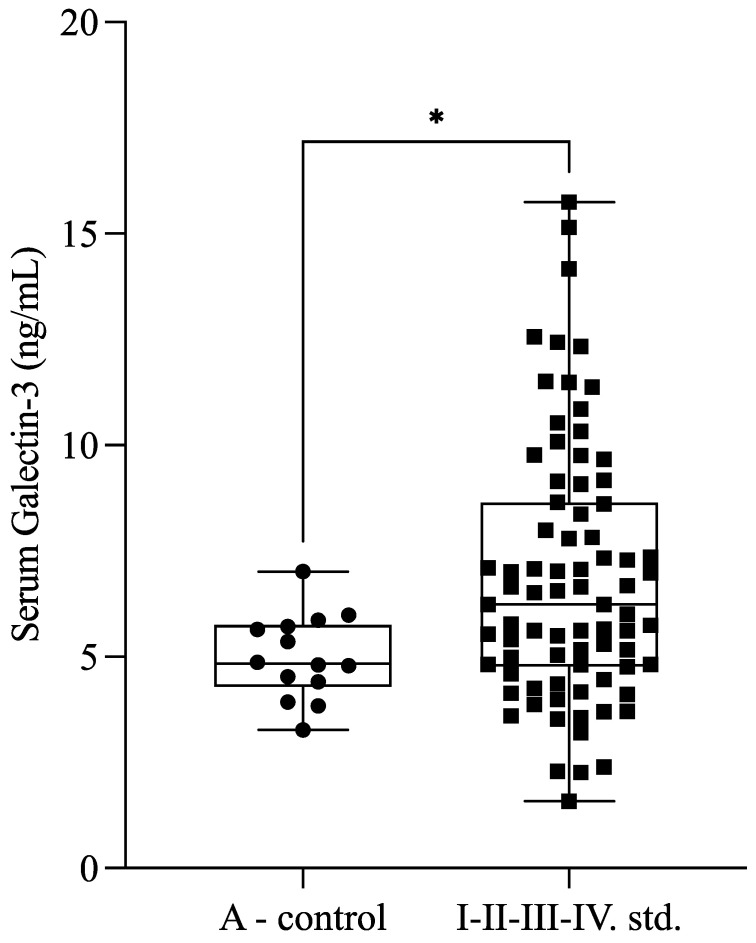
Serum galectin-3 levels in patients with endometriosis (stage I–IV) and healthy controls (A-control). The box plot diagram represents the interquartile range and median values. Whiskers indicate the most extreme observations. The individual values are presented with black dots (A-control group, *n* = 14) and squares (endometriosis patients, *n* = 77). The Mann–Whitney U test was used for statistical analysis. * *p* ≤ 0.05.

Serum Gal-3 levels were significantly higher (*p* = 0.030) in patients with endometriosis (*n* = 77; 6.65 ± 2.76 ng/mL) compared with healthy controls (*n* = 14; 4.99 ± 0.99 ng/mL) (Figure 1).

**Figure 2:**
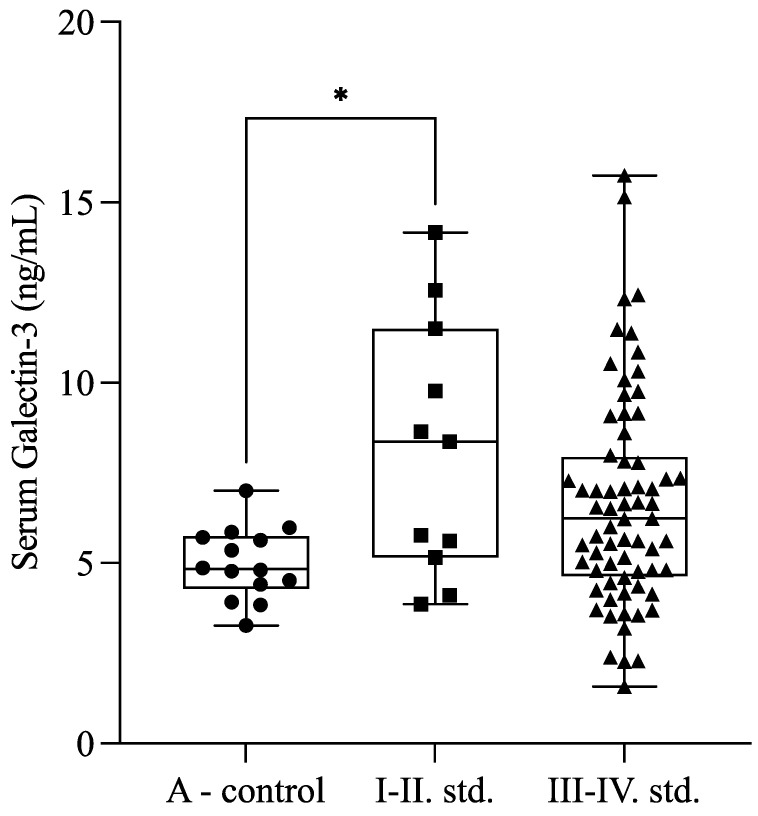
Serum galectin-3 levels in the healthy control group (A-control) and the minimal or mild (I–II) and severe (III–IV) stages of endometriosis. The box plot diagram represents the interquartile range and median values. Whiskers indicate the most extreme observations. The individual values are presented with black dots (A-control, *n* = 14), squares (minimal or mild endometriosis (I–II stage), *n* = 11), and triangles (severe endometriosis (III–IV stage) *n* = 66). The Kruskal–Wallis test with Dunn’s multiple comparisons was used for statistical analysis. * *p* ≤ 0.05.

Our results showed higher serum Gal-3 levels in the minimal and mild (I–II) stages of endometriosis compared with healthy controls (*p* = 0.041). Moreover, there was a tendency (*p* = 0.053) between minimal or mild (I–II; *n* = 11) and severe (III–IV; *n* = 66) stages of endometriosis. The Gal-3 levels were 8.14 ± 3.54 ng/mL in the mild endometriosis group (stages I–II), 6.40 ± 2.56 ng/mL in the severe endometriosis group (stages III–IV), and 4.99 ± 0.99 ng/mL in the control group (Figure 2).

We further investigated the differences between the follicular and secretory phases of the menstrual cycle, but we found no significant variation in levels between these phases. The Gal-3 level in the follicular phase (*n* = 40) was 6.35 ± 2.69 ng/mL, and that in the secretory phase (*n* = 37) was 5.91 ± 2.41 ng/mL (*p* > 0.05).

**Figure 3:**
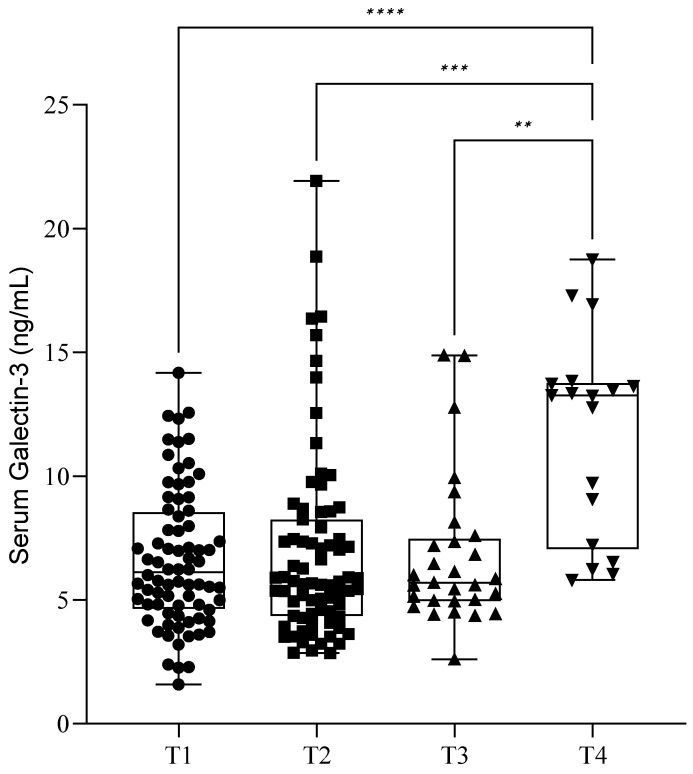
Serum galectin-3 levels during the follow-up of our patients after surgery. The box plot diagram represents the interquartile range and median values. Whiskers indicate the most extreme observations. The individual values are presented with black dots (before surgery (T1), *n* = 77), squares (after surgery (T2), *n* = 75), upward triangles (after one month (T3), *n* = 29), and downward triangles (after one year (T4), *n* = 18). Mixed-effects analysis with Geisser–Greenhouse correction and Holm–Sídák multiple comparison tests, with individual variances computed for each comparison, were used for statistical analysis. ** *p* ≤ 0.01, *** *p* ≤ 0.001, **** *p* ≤ 0.0001.

**Figure 4:**
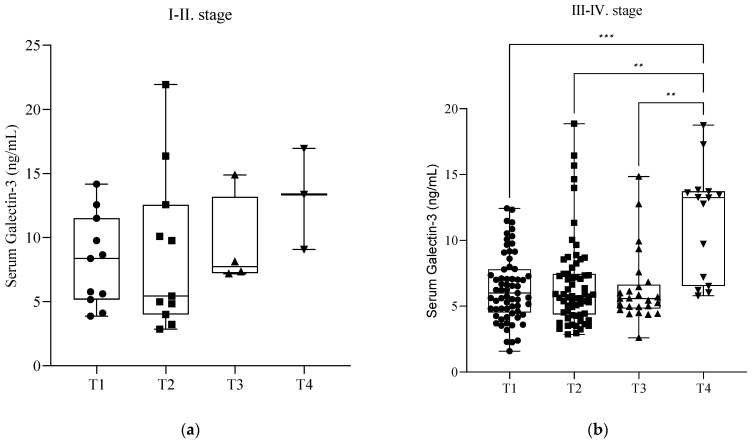
(**a**) Serum galectin-3 levels in patients with minimal or mild endometriosis (stage I–II) during follow-up after surgery. The box plot diagram represents the interquartile range and median values. Whiskers indicate the most extreme observations. The individual values are presented with black dots (before surgery (T1), *n* = 11), squares (after surgery (T2), *n* = 11), upward triangles (after one month (T3), *n* = 4), and downward triangles (after one year (T4), *n* = 3). Mixed-effects analysis with Geisser–Greenhouse correction and Holm–Sídák multiple comparison tests, with individual variances computed for each comparison, were used for statistical analysis. (**b**) Serum galectin-3 levels in patients with severe endometriosis (stage III–IV) during follow-up after surgery. The box plot diagram represents the interquartile range and median values. Whiskers indicate the most extreme observations. The individual values are presented with black dots (before surgery (T1), *n* = 66), squares (after surgery (T2), *n* = 64), upward triangles (after one month (T3), *n* = 25), and downward triangles (after one year (T4), *n* = 15). Mixed-effects analysis with Geisser–Greenhouse correction and Holm–Sídák multiple comparison tests, with individual variances computed for each comparison, were used for statistical analysis. ** *p* ≤ 0.01, *** *p* ≤ 0.001.

During the follow-up of our patients, we checked the serum soluble Gal-3 levels immediately before (T1) and one day after surgery (T2). The mean (SD) Gal-3 level before surgery (T1) was 6.64 ± 2.77 ng/mL, and it was 6.97 ± 3.89 ng/mL after surgery (T2). Of the 77 patients, 29 returned after one month (T3) for control blood sampling, and their Gal-3 value was 6.75 ± 3.00 ng/mL. Only 18 patients were present after one year (T4), and they showed an elevated lectin value of 11.73 ± 4.11 ng/mL. There was a significant elevation of serum Gal-3 levels between T1, T2, T3, and T4 blood sampling (*p* < 0.05) (Figure 3). Further investigation revealed that these significant elevations occurred only in the severe stages (III–IV) of endometriosis (*p* < 0.05) (Figure 4b). In the cases of mild endometriosis (I–II), we did not find any significant elevation of serum Gal-3 levels (Figure 4a). However, we cannot exclude that this could be due to the small number of elements.

**Figure 5:**
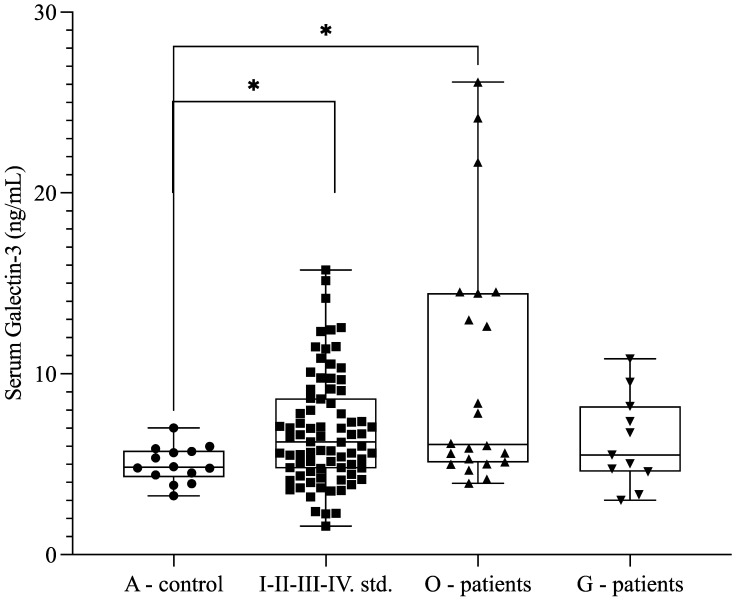
Serum galectin-3 levels in the control group (A-control), patients with endometriosis patients (stages IIV. stages), patients with endometrium-related malignant tumors (O-control), and patients with benign gynecological disease (G-patients). The box plot diagram represents the interquartile range and median values. Whiskers indicate the most extreme observations. The individual values are presented with black dots (A-control group, *n* = 14), squares (endometriosis group, *n* = 77), upward triangles (O-control, *n* = 22), and downward triangles (G-patients, *n* = 11). Kruskal–Wallis test with Dunn’s multiple comparison analysis was used for statistical analysis. * *p* ≤ 0.05.

Because of previously published data describing Gal-3 as an indicator of clear cell carcinomas, we measured the Gal-3 levels of patients with malignant (O-patients, low-grade endometrioid carcinoma) and benign gynecological tumors (G-patients, uterine and ovarian fibroids) at our tertiary referral center. We also observed higher soluble Gal-3 levels in patients with malignant diseases (*n* = 22; 9.99 ± 6.76 ng/mL) compared with the control group (*n* = 14; 4.99 ± 0.99 ng/mL) (*p* = 0.020) (Figure 5). The Gal-3 level was 6.267 +/− 2.514 ng/mL in patients with benign gynecologic conditions, which was comparable to the levels detected in patients with endometriosis (*p* > 0.05). Furthermore, there was an increasing trend between endometriosis (*n* = 77; 6.65 ± 2.76 ng/mL) and the O-patient group (*n* = 22; 9.99 ± 6.76 ng/mL) (*p* = 0.085) (Figure 5).

**Figure 6:**
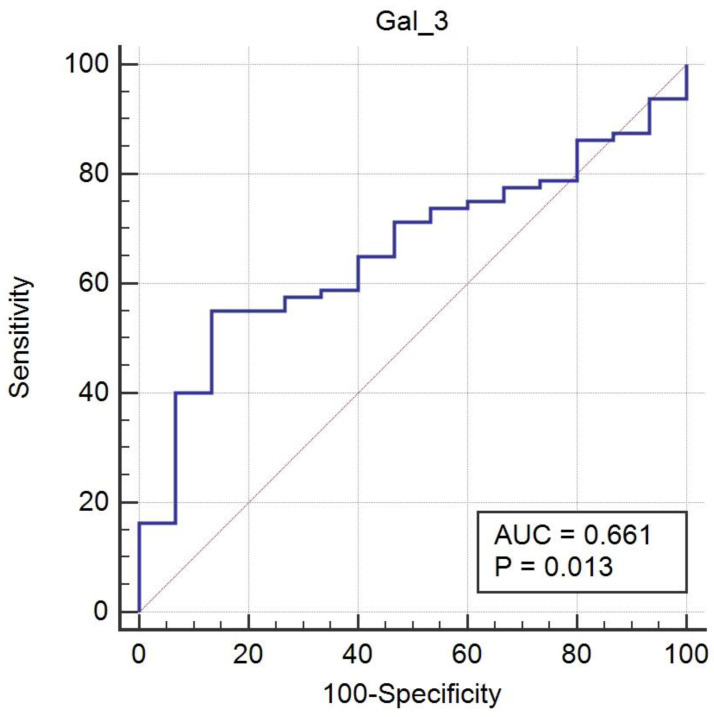
The receiver operating characteristic curve analysis of the serum Galectin-3 levels in patients with endometriosis and healthy controls.

ROC curve analysis indicated limited diagnostic value for serum Gal-3 levels in endometriosis diagnosis (*p* = 0.013), with an AUC of 0.661 (95% confidence interval, 0.556–0.755; Figure 6). The optimal cutoff point of 5.985 ng/mL, determined using Youden’s index, showed a sensitivity of 55% and specificity of 86.67%. With a 15% prevalence, the positive predictive value was 42.1%, and the negative predictive value was 91.6%. The diagnostic accuracy was 81.92%, suggesting lower diagnostic potential compared with our previous publication on serum Gal-9 [7].

## 3. Discussion

In patients with negative imaging, definitive endometriosis diagnosis requires laparoscopic intervention and histological analysis involving invasive evaluation and direct lesion visualization [8]. The initiation of endometriosis treatment often relies on a clinical hypothesis derived from observed signs and symptoms, which can contribute to significant delays in diagnosis. The absence of reliable non-invasive diagnostic tools results in an average diagnostic delay of 11.7 years in the United States, 8 years in the United Kingdom, and 3.9 years in Hungary [9,10]. Diagnosing endometriosis in adolescents can be challenging due to the non-cyclic nature of pain symptoms, leading to an average delay of 7 years between symptom onset and diagnosis [11]. Non-invasive diagnostic tools are crucial for the early diagnosis of endometriosis, particularly for minimal–mild (ASRM Stage I–II) cases [8]. As more women could benefit from earlier surgical intervention or undergo oocyte preservation, fewer would be reproductively challenged. The criteria for an ideal diagnostic biomarker are that it is non-invasive, modestly priced, highly accurate, and reproducible outside research conditions. Furthermore, the biomarker should be suitable to distinguish between disease stages, respond to treatment, and react to disease progression [12].

Our study revealed significantly higher serum Gal-3 levels in patients with endometriosis compared with the asymptomatic control group. In contrast to the previous findings—where patients with severe endometriosis had higher Gal-3 levels than patients with mild endometriosis [13]—our results showed that stage I–II patients had higher Gal-3 levels than stage III–IV patients. Gal-1, 3, and 9 are associated with neoplasm development, particularly in gynecological cancers [14]. Under normal physiological conditions, galectins regulate the cell cycle and prevent tumor formation; disrupted galectin function may promote cancer development and metastasis [15]. It was previously shown that Gal-3 might lead to infertility in patients with endometriosis-related conditions because of progesterone resistance [16]. Gal-3 also regulates NK-kB signaling in clear cell carcinoma, a common form of endometriosis-associated ovarian cancer (EAOC). This indicates that, at least in higher-stage tumors, Gal-3 may be a possible prognostic marker for EAOC [17]. These previous findings correlate with our results, showing a significantly higher level of Gal-3 in patients with endometrium-related malignant transformations than in patients with or without endometriosis, and they suggest its potential differential diagnostic value in cases of endometrium-related malignancies. Endometriosis is a benign disease, but on the other hand, the cellular and molecular characteristics of the infiltrative form of endometriosis, along with its potential to spread to other tissues, resemble malignancy [18,19], which could be the reason for the elevated levels in endometriosis, similar to malignancies.

The lack of diagnostic biomarkers for endometriosis poses a significant challenge, as it hinders the ability to identify the condition accurately and efficiently. The absence of reliable biomarkers makes it challenging to establish an early diagnosis and initiate appropriate treatment, potentially leading to the development of chronic pain syndromes in the long term [20]. The delay in diagnosis and subsequent treatment can negatively affect adolescents with endometriosis. Prolonged exposure to pain can sensitize the nervous system and increase the likelihood of developing chronic pelvic pain and other chronic pain syndromes [21]. Early recognition and management of endometriosis in adolescents are crucial to mitigate the impact of chronic pain and improve long-term outcomes [20]. Efforts are needed to improve awareness among healthcare professionals about the atypical presentations of endometriosis in adolescents and the importance of early diagnosis.

Despite the relatively small sample size, our study’s patient cohort was consistent with sample sizes used in previous similar research [7]. The alterations in serum Gal-3 levels during follow-up after the surgery need further investigation since we could not explain the observed changes in serum Gal-3 levels. We need to test our hypothesis on independent datasets due to this limitation. Furthermore, a clinically important question remains open. Even if we succeeded in removing all visible and invisible lesions based on a reliable, non-invasive test, is there evidence that early diagnosis may improve future fertility or quality of life? According to our data, Gal-3-based non-invasive testing is a promising tool for the early diagnosis of endometriosis. The obvious next step will be to prospectively study the clinical value of the Gal-3 test in larger and more representative groups of patients.

## 4. Materials and Methods

### 4.1. Patients and Sample Collecting

The Institutional Ethical and Review Board of Semmelweis University, Budapest, approved the study protocol in accordance with the Declaration of Helsinki to protect the rights of human participants (registration no. 143/2008).

Informed consent was obtained from all participants prior to their involvement in this prospective case–control study. Between 1 September 2021 and 1 September 2023, participants were chosen successively (Clinical trial identifier: NCT04401592). Laparoscopy was performed on 77 women of reproductive age investigated for pelvic pain and/or infertility. The demographic data are presented in Supplementary Table S1.

Laparoscopic surgery was performed on 11 patients due to benign gynecological diseases, such as uterine (*n* = 4) and ovarian fibroids (*n* = 7) (Supplementary Table S2), while 22 patients were included in our study due to surgical intervention for oncologic disorders (Supplementary Table S3). All of the oncologic patients had low-grade (grade 1 and grade 2) endometrioid carcinoma.

In our endometriosis group, women were classified into minimal–mild (stage I–II) (*n* = 11) and moderate–severe (stage III–IV) (*n* = 66) disease categories according to the ASRM scoring system (Revised American Society for Reproductive Medicine classification of endometriosis, 1997). Endometriosis was confirmed histologically from the tissue biopsies taken during surgical intervention by the Department of Pathology and Experimental Cancer Research, Semmelweis University [7].

**Figure 7:**
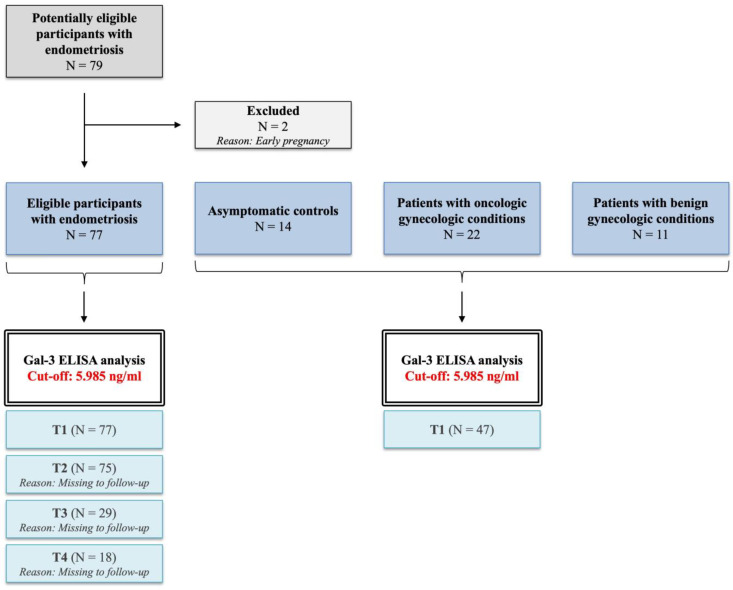
Flowchart of study population.

For Gal-3 ELISA, serum samples were collected one day before (T1 group, *n* = 77) and after the laparoscopy (T2 group, *n* = 75), and then one month (T3 group, *n* = 29) and one year (T4 group, *n* = 18) later during patient follow-up. These patient groups (T1, T2, T3, and T4) were derived from the same cohort and investigated linearly during follow-up. The inclusion criteria were patients between the ages of 15–40 with no current hormonal medical treatment. Patients with gynecologic malignancies were included, as stated above. Ongoing pregnancy was the only exclusion criterion (Figure 7).

After obtaining written informed consent, the control group was built anonymously from age-matched, healthy female blood donors (*n* = 14) with the permission of the National Blood Bank Regional Center Budapest. The study did not include women who mentioned gynecologic complaints. Demographic data for the participants were not available. The blood samples were allowed to coagulate at room temperature and then centrifuged for 10 min at 3000 rpm to separate the cellular components. The serum was then aliquoted and stored at −80 °C until further analysis using ELISA. All serum samples were thawed only once before the ELISA measurements [7].

### 4.2. Galectin-3 ELISA

To determine the levels of soluble Gal-3 in the serum of women with endometriosis or gynecological tumors as well as healthy controls, a human Gal-3-specific quantitative sandwich ELISA was used (Quantikine, human Gal-3 ELISA kit, R&D Systems, Minneapolis, MN, USA; Cat. no: DGAL30) following the manufacturer’s protocol.

The optical density (OD) was measured at 450 nm with a 540 nm wavelength correction using a SPECTROStar Nano microplate reader (BMG Labtech, Ortenberg, Germany). The Gal-3 concentrations were calculated from the standard curve by comparing the sample OD values to the standard ODs, as the OD values were proportional to the Gal-3 levels in the test samples. The concentration of soluble Gal-3 in the samples was determined using MARS Data Analysis Software version 3.32 (BMG Labtech, Ortenberg, Germany), which applied a 4-parametric logistic analysis. The obtained data are presented in ng/mL [7]. The test sensitivity was 0.003–0.085 ng/mL. Minimum detectable dose (MDD): 0.016 ng/mL. Range of the standard curve: 0–10 ng/mL. Intra-assay precision (CV%) for serum/plasma samples: 3.5–3.8%, Inter-assay precision: 5.8–6.3%.

### 4.3. Statistical Analysis

GraphPad Prism version 9.5.0 (GraphPad Software, San Diego, CA, USA) was used for statistical analysis. ROC analysis was performed with MedCalc software version 16.8 (MedCalc Software bvba). The study data were evaluated using descriptive statistical methods, including mean, standard deviation (SD), and distribution. Outliers were identified and removed using the robust regression and outlier removal (ROUT) method. The appropriate statistical tests were used to compare the groups on the basis of the number of groups, data distribution, and variable types. This included the Unpaired *t*-test, Mann–Whitney U test, Kruskal–Wallis test with Dunn’s comparison, and mixed-effects analysis with Holm–Sídák multiple comparison test. For groups with normally distributed data but varying homogeneity of variance, Geisser–Greenhouse correction was applied to ensure accurate comparisons and avoid potential biases in the results. Differences were considered statistically significant if the *p*-value was less than or equal to 0.05.

The ROC curve was used to determine the diagnostic performance of Gal-3 ELISA for endometriosis. Gal-3 values from confirmed endometriosis samples were used as the positive group, while the serum of healthy women served as the negative samples. Youden’s index was used to select the optimal cut-off point. The calculated AUC was considered significantly different from the null hypothesis if its value exceeded 0.5 [7].

## Data Availability

The datasets used and/or analyzed during this study are available from the corresponding author upon request.

## Supplementary Materials

The following supporting information can be downloaded at: https://www.mdpi.com/article/10.3390/ijms26041630/s1.
