# Supplementary material for: Alteration of Serum Gal-3 Levels in Endometrium-Related Reproductive Disorders
Source: Int J Mol Sci. 2025 Feb 14;26(4):1630. doi: 10.3390/ijms26041630 (PMC11855016; doi:10.3390/ijms26041630)
Supplement: Supplementary file 1 [file ijms-26-01630-s001.zip › ijms-3420931-supplementary.pdf]

Article

Alteration of serum Gal-3 levels in endometrium-related reproductive disorders

Reka Brubel[1*,], Beata Polgar[2], Laszlo Szereday[2], Dora Bianka Balogh[1], Tunde Toth[3], Szabolcs Mate[1], Noemi Csibi[1], Noemi Dobo[1], Gernot Hudelist[4], Nandor Acs[1] and Attila Bokor[1].

Supplemental Table 1

Scheme 1. Demographic characteristics of the Study Population.

	Endometriosis Std I.-II. (*n* = 11)	Endometriosis Std III.-IV. (*n* = 66)
Age (years, mean ± SD)	31.2 ± 4.3	34.9 ± 5.2
Infertility [*n* (%)]	7 (63.6%)	44 (66.67%)
Previous Surgery [*n* (%)]	3 (27.27%)	42 (63.6%)
CPP [*n* (%)]	11 (100%)	66 (100%)
Autoimmun Disease* [*n* (%)]	2 (18.18%)	5 (7.58%)
Insuline resistance [*n* (%)]	1 (9.09%)	6 (9.09%)
Follicular [*n* (%)]	5 (45.45%)	35 (53.03%)
Luteal [*n* (%)]	6 (54.55%)	31 (46.96%)

* Hypothyreosis, Ceoliakia, Chron-desease

Supplemental Table 2

Scheme 2. Demographic characteristics of the Study Population.

	Benign gynecologic patients (*n* = 11)
Age (years, Mean)	66.6 ± 8.2
Previous Pregnancy [*n* (%)]	8(72.72%)
Previous Surgery [*n* (%)]	5(45.45%)
Autoimmun Disease* [*n* (%)]	1(9.09%)
Insuline resistance [*n* (%)]	0 (0%)
Menopause [*n* (%)]	5 (45.45%)
Other Disease * * [*n* (%)]	7 (63.63%)

* Hypothyreosis, Ceoliakia, Chron-desease
* * Hypertension, Type II Diabetes Mellitus

Supplemental Table 3

Scheme 3. Demographic characteristics of the Study Population.

	Oncologic patients (n=22)
Age (years, Mean)	62.6
Previous Pregnancy [n(%)]	11(50%)
Previous Surgery [n(%)]	22(100%)
Autoimmun Disease* [n(%)]	0(0%)
Insuline resistance	1 (4,5%)
Menopause [n(%)]	20 (90,9%)
Other Disease * * [n(%)]	11(50%)

* Hypothyreosis, Ceoliakia, Chron-desease
* * Hypertension, Type II Diabetes Mellitus
